# Prevalence, functional characteristics, and clinical significance of right ventricular involvement in patients with hypertrophic cardiomyopathy

**DOI:** 10.1038/s41598-020-78945-4

**Published:** 2020-12-14

**Authors:** Jiwon Seo, Yoo Jin Hong, Young Jin Kim, Purevjargal Lkhagvasuren, Iksung Cho, Chi Young Shim, Jong-Won Ha, Geu-Ru Hong

**Affiliations:** 1 Cardiology Division, Severance Cardiovascular Hospital, Yonsei University College of Medicine, 50-1 Yonsei-ro, Seodaemun-gu, Seoul, 03722 Republic of Korea; 2 Department of Radiology, Research Institute of Radiological Science, Severance Hospital, Yonsei University College of Medicine, Yonsei University Health System, Seoul, Republic of Korea

**Keywords:** Cardiology, Diseases

## Abstract

We sought to investigate the prevalence, functional characteristics, and clinical significance of right ventricular (RV) involvement in patients with hypertrophic cardiomyopathy (HCM). A total of 256 patients with HCM who underwent both cardiac magnetic resonance (CMR) imaging and transthoracic echocardiography within 6 months of each other were retrospectively analysed. RV involvement was defined as an increased RV wall thickness ≥ 7 mm on CMR in the segments of the RV free wall. Primary outcomes were defined as the composite of all-cause death, heart transplantation, and unplanned cardiovascular admission. Thirty-seven (14.4%) patients showed RV involvement. Patients with RV involvement showed a significantly higher left ventricular (LV) maximal wall thickness and left atrial volume index. Multivariate Cox model revealed that RV involvement was independently associated with primary outcomes (HR: 2.30, p = 0.024). In a subgroup analysis of patients with speckle tracking echocardiography (n = 190), those with RV involvement had significantly more impaired RV strain, which was independently associated with primary outcomes. RV involvement in patients with HCM correlated with more advanced LV structure and biventricular dysfunction, suggesting an indicator of severe HCM. RV involvement and impaired RV strain have a prognostic value related to clinical adverse events in patients with HCM.

## Introduction

Many studies on hypertrophic cardiomyopathy (HCM) have focused on the left ventricle (LV). Maximal left ventricular wall thickness, left ventricular outflow tract (LVOT) obstruction, LV apical aneurysm, and late gadolinium enhancement (LGE) on cardiac magnetic resonance (CMR) imaging are suggested risk factors for sudden cardiac death (SCD) in HCM^1–3^.

On the other hand, the right ventricle (RV) relatively has long been neglected, perhaps because RV is not considered a major risk factor for SCD related to HCM, and it is difficult to measure RV thickness accurately by transthoracic echocardiography (TTE). A few studies have recently reported the prevalence and clinical significance of RV involvement using CMR imaging^4,5^ and clinical/subclinical RV dysfunction in patients with HCM^6,7^. Although these results suggest that the presence or absence of RV involvement may be important for the current disease status and clinical prognosis in patients with HCM, the prevalence, structural and functional characteristics, and prognostic significance of RV involvement in patients with HCM are still ambiguous. Therefore, the objectives of this study were to identify the prevalence of RV involvement in patients with HCM using CMR imaging and to investigate whether RV involvement and RV dysfunction have prognostic significance in determining clinical outcomes.

## Results

### Prevalence and baseline characteristics

Among the 256 patients with HCM, 37 (14.4%) with RV involvement were identified by CMR. Substantial interobserver agreement was achieved for the evaluation of RV involvement (kappa value, 0.89). Baseline characteristics of the study population are presented in Table 1. Patients’ mean age was 53.1 ± 14.2 years, 180 (70.3%) were men, and 42 (16.4%) were diagnosed with atrial fibrillation (AF). Patients with RV involvement had a higher percentage of AF and had more frequently received an implantable cardioverter defibrillator (ICD) than those without RV involvement. Patients with RV involvement showed a significantly higher LVMWT, higher left atrial volume index (LAVI) and higher E/e’ than those without RV involvement. CMR imaging data showed that the LVMWT and LV end systolic volumes were significantly higher in patients with RV involvement than in those without. RV LGE was also predominantly detected in patients with RV involvement.

**Table 1:** Baseline characteristics of the study population.

	Total patients (n = 256)	No RV involvement (n = 219)	RV involvement (n = 37)	P value*
**Demographic and clinical data**				
Age, year	53.1 ± 14.2	53.4 ± 14.3	51.5 ± 14.1	0.454
Male sex, n (%)	180 (70.3)	150 (68.8)	29 (78.4)	0.334
BMI, kg/m^2^	24.8 ± 3.2	24.9 ± 3.2	24.1 ± 3.3	0.153
Hypertension, n (%)	189 (73.8)	164 (74.9)	25 (67.6)	0.463
Diabetes mellitus, n (%)	48 (18.8)	40 (18.3)	8 (21.6)	0.798
Dyslipidemia, n (%)	87 (34.0)	70 (32.0)	17 (45.9)	0.141
Atrial fibrillation, n (%)	42 (16.4)	30 (13.7)	12 (32.4)	0.009
Family history of SCD, n (%)	40 (15.6)	33 (15.1)	7 (18.9)	0.725
ICD, n (%)	21 (8.2)	13 (5.9)	8 (21.6)	0.004
Syncope, n (%)	34 (13.3)	25 (11.4)	9 (24.3)	0.060
Beta blocker, n (%)	127 (49.6)	110 (50.2)	17 (45.9)	0.761
Calcium channer blocker, n (%)	54 (21.1)	43 (19.6)	11 (29.7)	0.240
RAS blocker, n (%)	111 (43.4)	102 (46.6)	9 (24.3)	0.019
Aspirin, n (%)	62 (24.2)	49 (22.4)	13 (35.1)	0.142
Statin, n (%)	83 (32.4)	65 (29.7)	17 (45.9)	0.077
**Echocardiographic data**				
LVEF, %	68.6 ± 8.7	68.9 ± 8.2	66.8 ± 11.0	0.277
LAVI, mL/m^2^	41.6 ± 15.9	40.3 ± 14.8	49.1 ± 20.0	0.014
RV systolic pressure, mm Hg	27.0 ± 7.0	26.6 ± 6.6	29.3 ± 8.8	0.080
E/e’	15.4 ± 7.0	15.1 ± 7.1	17.5 ± 6.5	0.051
LV maximal thickness, mm	21.2 ± 4.7	20.6 ± 4.3	24.6 ± 5.2	< .001
RV thickness, mm	4.4 ± 1.4	4.1 ± 0.9	5.8 ± 2.5	< .001
LVOT obstruction, n (%)	62 (24.2)	52 (23.7)	10 (27.0)	0.823
**Cardiac MR data**				
LV maximal thickness, mm	21.6 ± 5.2	21.2 ± 5.0	23.8 ± 5.7	0.006
RV maximal thickness, mm	5.4 ± 1.8	4.8 ± 0.6	9.0 ± 2.2	< .001
LVEDV, ml	143.8 ± 34.2	143.6 ± 33.3	144.9 ± 39.6	0.842
LVESV, ml	48.1 ± 19.7	46.9 ± 19.3	55.6 ± 21.2	0.017
LVEF, %	67.5 ± 8.8	68.3 ± 8.2	62.6 ± 10.3	< .001
RVEDV, ml	131.3 ± 66.5	131.8 ± 70.2	128.4 ± 37.9	0.681
RVESV, ml	50.8 ± 20.5	50.8 ± 20.3	50.9 ± 21.5	0.983
RVEF, %	61.2 ± 8.9	61.2 ± 8.7	61.0 ± 10.5	0.896
LV LGE, n (%)	236 (92.2)	199 (90.9)	37 (100.0)	0.113
RV LGE, n (%)	26 (10.2)	5 (2.3)	21 (56.8)	< .001

*RV* right ventricle, *LV* left ventricle, *BMI* body mass index, *SCD* sudden cardiac death, *RAS* renin–angiotensin system, *LVEF* LV ejection fraction, *LAVI* left atrial volume index, *LVOT* left ventricular outflow tract, *LS* longitudinal strain, *GLS* global LS, *MR* magnetic resonance, *EDV* end diastolic volume, *ESV* end systolic volume, *LGE* late gadolinium enhancement.

*P value between patients with RV involvement and without RV involvement.

### Clinical outcomes

**Figure 1:**
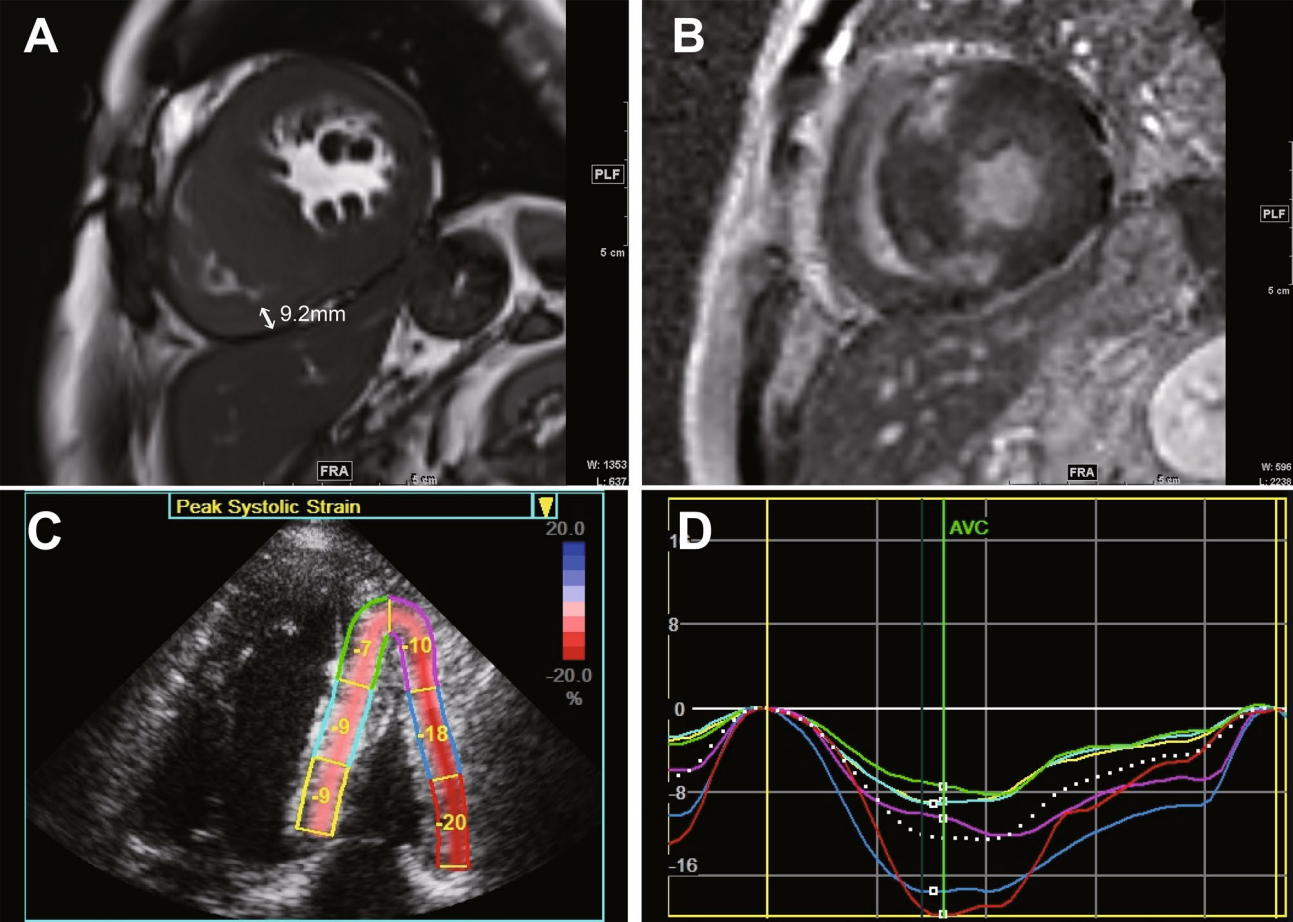
Representative case of HCM with RV involvement. **(A)** Significant hypertrophy of the RV apex (9.2 mm) and** (B)** late gadolinium enhancement in the anteroseptal and inferoseptal wall were seen. **(C,D)** Impaired right ventricular two-dimensional speckle-tracking strain pattern is seen in the patient.

During the follow-up period, there were 33 primary outcomes, including all-cause death (n = 3), heart transplantation (n = 1), and unplanned cardiovascular admission (n = 29). Two patients had sudden cardiac death, and one patient died from lung cancer. Among the 29 cardiovascular hospitalizations, 10 were admitted for heart failure, 5 had ongoing and recurrent angina, 5 were admitted for atrial tachyarrhythmia, 2 were admitted for ventricular tachyarrhythmia, 4 had a sudden collapse, and 3 had stroke. Table 2 shows univariate and multivariate Cox proportional hazard models for the primary outcomes in total patient population (n = 256). In univariate Cox proportional hazard analysis, the presence of AF, a history of unexplained syncope, increased LAVI, elevated RVSP, higher E/e’, and lower LVEF measured by CMR were significantly associated with a higher risk of primary outcomes. In multivariate analysis, the presence of AF, a history of unexplained syncope, higher E/e’, lower LVEF measured by CMR, and the presence of RV involvement were independently associated with primary outcomes. Figure 1A shows Kaplan–Meier curves for primary outcome-free survival according to the presence of RV involvement. There was a significantly higher probability of primary outcomes in patients with RV involvement (p < 0.001). In a subgroup analysis of 190 patients with analysable speckle tracking echocardiography, 79 (41.6%) had impaired RV free wall LS (> − 20%) as shown in Table 3. Impaired RV free wall LS (hazard ratio [HR] = 3.07, 95% confidence interval [CI] = 1.16–8.09, p = 0.023), higher E/e’, and lower LVEF were independent factors associated with primary outcomes (Table 4). Kaplan–Meier curves for primary outcome-free survival, according to RV free wall LS, revealed that the most deleterious primary outcomes were in patients with impaired RV free wall LS, as shown in Fig. 1B.

**Table 2:** Univariate and multivariate Cox proportional hazard models for primary outcomes in total study population (n = 256).

Variables	Univariate		Multivariate	
	HR (95% CI)	P value	HR (95% CI)	P value
Age	1.02 (0.99–1.05)	0.172		
Male sex	0.72 (0.35–1.47)	0.367		
Atrial fibrillation	4.45 (2.24–8.83)	< .001	2.25 (1.01–5.01)	0.048
Familiar history of SCD	0.32 (0.08–1.33)	0.117		
Syncope	2.55 (1.18–5.50)	0.017	2.31 (1.05–5.15)	0.038
LAVI	1.03 (1.01–1.04)	0.002	1.00 (0.98–1.02)	0.906
RVSP	1.08 (1.03–1.13)	0.001	1.02 (0.97–1.07)	0.479
E/e’	1.05 (1.02–1.08)	< .001	1.05 (1.01–1.09)	0.012
LVOT obstruction	0.93 (0.42–2.07)	0.858		
LVEF	0.93 (0.89–0.96)	< .001	0.94 (0.91–0.98)	0.005
RV involvement	4.13 (2.05–8.32)	< .001	2.30 (1.10–4.82)	0.024
LV maximal wall thickness	0.97 (0.90–1.05)	0.477		
LVESV	1.01 (0.99–1.03)	0.311		
RV LGE	2.06 (0.89–4.77)	0.091		

*SCD* sudden cardiac death, *LAVI* left atrial volume index, *RVSP* right ventricular systolic pressure, *LVOT* left ventricular outflow tract, *RV* right ventricle, *LV* left ventricle, *LVEF* LV ejection fraction, *CMR* cardiac magnetic resonance, *LVESV* left ventricular end systolic volume, *LGE* late gadolinium enhancement.

**Table 3:** Comparison of the left and right ventricular longitudinal strain values between patients with RV involvement and those without RV involvement (n = 190).

Longitudinal strain	Total patients (n = 190)	No RV involvement (n = 160)	RV involvement (n = 30)	P-value*
LV GLS, %	− 11.7 ± 4.8	− 12.2 ± 4.9	− 9.5 ± 3.3	0.001
RV GLS, %	− 18.6 ± 5.5	− 19.4 ± 5.3	− 14.5 ± 5.0	< .001
RV septal wall LS, %	− 12.9 ± 6.4	− 13.5 ± 6.1	− 10.0 ± 7.2	0.006
RV free wall LS, %	− 21.8 ± 7.1	− 22.9 ± 6.9	− 16.4 ± 5.1	< .001
RV free wall LS > − 20%, n (%)	79 (41.6%)	55 (34.6%)	24 (77.4%)	< .001

*RV* right ventricle, *LV* left ventricle, *LS* longitudinal strain, *GLS* global LS.

*P value between patients with RV involvement and patients without RV involvement.

**Table 4:** Univariate and multivariate Cox proportional hazard model for primary outcomes in patients with strain echocardiography (n = 190).

Variables	Univariate		Multivariate	
	HR (95% CI)	P value	HR (95% CI)	P value
Age	1.01 (0.98–1.04)	0.622		
Male sex	0.58 (0.26–1.27)	0.173		
Atrial fibrillation	5.85 (2.37–12.85)	< .001	2.15 (0.76–6.12)	0.151
Syncope	2.80 (1.16–6.74)	0.022	4.03 (1.54–10.56)	0.005
Familiar history of SCD	0.21 (0.03–1.58)	0.131		
LAVI	1.03 (1.01–1.05)	0.010	0.99 (0.99–1.02)	0.245
RVSP	1.08 (1.03–1.13)	0.003	1.06 (0.99–1.13)	0.077
E/e’	1.06 (1.02–1.09)	< .001	1.05 (1.00–1.11)	0.037
Obstructive type	0.88 (0.39–2.00)	0.761		
LVEF	0.93 (0.89–0.97)	< .001	0.95 (0.91–0.99)	0.022
RV free wall LS > − 20%	4.03 (1.68–9.67)	0.002	3.07 (1.16–8.09)	0.023
LV maximal thickness	0.97 (0.90–1.05)	0.477		
LVESV	1.01 (0.99–1.02)	0.630		
RV LGE	1.82 (0.68–4.88)	0.232		

*SCD* sudden cardiac death, *LAVI* left atrial volume index, *RVSP* right ventricular systolic pressure, *RV* right ventricle, *LV* left ventricle, *LVEF* left ventricular ejection fraction, *CMR* cardiac magnetic resonance, *LVESV* left ventricular end systolic volume, *LGE* late gadolinium enhancement.

### RV dysfunction

**Figure 2:**
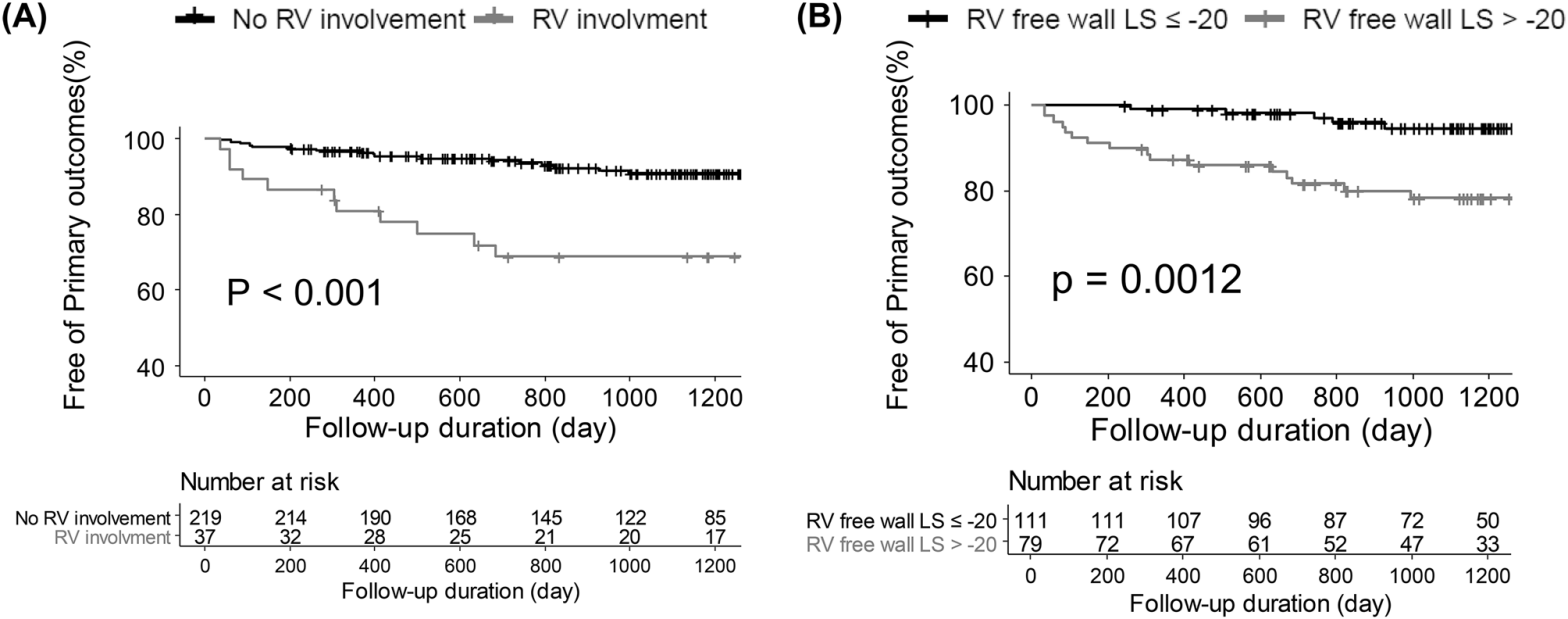
Kaplan–Meier curves for primary outcome-free survival **(A)** according to the presence of right ventricular involvement and **(B)** according to the presence of impaired right ventricular free wall longitudinal strain.

In a subgroup analysis, 190 patients with speckle tracking echocardiography were analysed separately to compare RV mechanical function and its association with RV involvement. Supplementary Table 1 shows baseline characteristics of the 190 patients. Patients with RV involvement had more impaired LV GLS, RV GLS, RV septal wall LS, and RV free wall LS than those without RV involvement. RV GLS (R = 0.345, p < 0.001) and RV free wall LS (R = 0.310, p < 0.001) were significantly correlated with RV maximal wall thickness measured on CMR imaging (see Fig. 2A,B). In multivariate logistic regression analysis for echocardiographic parameters associated with RV involvement, RV free wall LS (odds ratio [OR] = 1.01, 95% CI = 1.00–1.01, p = 0.046), RV thickness (OR = 1.09, 95% CI = 1.06–1.12, p < 0.001), LV GLS (OR = 1.01, 95% CI = 1.00–1.03, p = 0.034), and LVMWT (OR = 1.01, 95% CI = 1.00–1.02, p = 0.010) were independently associated with RV involvement (Table 5). Moreover, RV GLS and RV free wall LS had incremental values for the prediction of RV involvement in C-statistics. RV wall thickness with RV GLS (AUC = 0.813) and RV wall thickness with RV free wall LS (AUC = 0.816) demonstrated better correlation with RV involvement than only using RV wall thickness measured using echocardiography (AUC = 0.727), as shown in Fig. 2C,D.

**Table 5:** Univariate and multivariate logistic regression analysis to estimate association between echocardiographic parameters and right ventricular involvement in hypertrophic cardiomyopathy.

Variables	Univariate		Multivariate	
	OR (95% CI)	P value	OR (95% CI)	P value
LAVI	1.01 (1.00–1.01)	0.008	1.00 (0.99–1.00)	0.179
RVSP	1.01 (0.99–1.01)	0.084		
E/e’	1.01 (1.00–1.02)	0.013	1.00 (0.99–1.01)	0.448
LVOT obstruction	1.05(0.93–1.19)	0.423		
LVEF	0.99 (0.99–1.00)	0.299		
LV GLS	1.03 (1.02–1.04)	< .001	1.01 (1.00–1.03)	0.034
RV GLS	1.02 (1.01–1.03)	< .001		
RV septal LS	1.02 (1.01–1.03)	< .001		
RV free wall LS	1.02 (1.01–1.02)	< .001	1.01 (1.00–1.01)	0.046
RV thickness	1.12 (1.08–1.15)	< .001	1.09 (1.06–1.12)	< .001
LV maximal thickness	1.03 (1.02–1.04)	< .001	1.01 (1.00–1.02)	0.010

*OR* odds ratio, *CI* confidence interval, *LAVI* left atrial volume index, *RVSP* right ventricular systolic pressure, *LVOT* left ventricular outflow tract, *LVEF* left ventricular ejection fraction, *LV* left ventricle, *RV* right ventricle, *GLS* global longitudinal strain, *LS* longitudinal strain.

## Discussion

The main findings of the study were as follows: (1) RV involvement in patients with HCM is common (14.4%); (2) patients with RV involvement showed more advanced biventricular dysfunction, suggesting an indicator of severe HCM; (3) RV involvement and impaired RV longitudinal strain in patients with HCM showed prognostic values related to clinical adverse events; and (4) impaired RV GLS and RV free wall LS were more frequently detected in patients with RV involvement than in those without.

Many previous studies on RV involvement in HCM were sporadic case reports of severe RV hypertrophy and RV outflow tract obstruction^8,9^, and only a few studies described the prevalence of RV involvement in HCM. In an early study, using transthoracic echocardiography, RV hypertrophy was reported in 44% of patients with HCM^10^. Studies using CMR demonstrated a prevalence ranging from 1.3 to 30%, depending on the criteria of RV involvement^4,5,11^. Our study showed a prevalence of 14.4%, with the RVMWT ≥ 7 mm on CMR. The varied reported prevalence of RV involvement in HCM is postulated to be a consequence of the different criteria of RV involvement and modalities measured.

Classically, a family history of HCM-related SCD, unexplained syncope, multiple and repetitive non-sustained ventricular tachycardia, massive LVH, LV apical aneurysm or burn out stage (EF < 50%), and extensive LGE were suggested risk factors for SCD in HCM^3,12^. LVOT obstruction, diastolic dysfunction, and atrial tachyarrhythmia were considered risk factors related to heart failure^13,14^. Consistent with prior studies, our results showed that diastolic dysfunction, AF, unexplained syncope, and reduced LVEF were related to adverse clinical events but LVOT obstruction was not. We assumed that patients with high risk of SCD were managed with optimal medical therapy, ICD implantation, or septal myectomy, which may be responsible for these results. The association between RV involvement and poor prognosis indicated that patients with RV involvement had more advanced HCM and presented with significantly higher LAVI, higher E/e’, lower LVEF, and more impaired LV GLS in this study. LAVI and E/e’ reflect the LV end-diastolic pressure and long-term effects of elevated LV filling pressures; they are well correlated with LV diastolic burden and poor prognosis in patients with HCM^15–17^. Moreover, abnormal LV GLS has been shown to occur in HCM and is associated with a worse prognosis, even in patients with a normal LV ejection fraction^18^. Therefore, it is reasonable to assume that RV involvement is associated with severe systolic and diastolic dysfunction in HCM, which may play a role in determining clinical outcomes.

Prior literature regarding RV in HCM has described RV structural features and dysfunction separately. Some studies revealed that the structural feature, RV hypertrophy, is associated with poor clinical outcomes. In addition, RV dysfunction is frequently observed in patients with HCM and is associated with an increased likelihood of adverse clinical event^7,19,20^. Recently, Wu et al. showed that RV hypertrophy exhibits more reduced RV GLS, supported exercise capacity, and can independently predict exercise intolerance in patients with HCM^6^. Xiang Li et al. also presented that impaired RV myocardial strain was more obvious in the presence of RVH and LGE in RV^21^. Our results supported these prior studies and showed both structural abnormality and functional impairment of RV using CMR imaging in a relatively large study population. Patients with RV involvement had more impaired RV mechanical function, and both RV involvement and impaired RV dysfunction were independently associated with clinical outcomes. Therefore, we assumed that RV involvement implies more severe myocardial dysfunction, not only of LV but also of RV.

Interestingly, our study showed a large discrepancy in RV thickness as determined using transthoracic echocardiography and RVMWT on CMR imaging. We assumed this is due to the differing measurement sites and methods. In transthoracic echocardiography, RV thickness was calculated as the diameter of the RV free wall below the tricuspid annulus at a distance approximating the length of the anterior tricuspid leaflet, as recommended in the current guidelines. In contrast, RVMWT on CMR imaging measured the greatest diameter of the segments of the RV free wall, including the basal, mid, and apical levels of the RV free wall, in this study. Considering the methodological difficulty in measuring RV thickness using echocardiography, impaired RV strain is a more meaningful predictor of the structural change in RV and clinically adverse events in patients with HCM.

This study has several limitations. First, the retrospective study design investigated a single-centre registry; thus, there is a possibility of selection bias. Considering LGE was observed in > 90% of patients in our study population, it is suggested that many patients with advanced HCM were selected in this study. Second, the criteria of RV involvement can be deemed arbitrary because standard criteria for RV involvement in HCM were not established. Maron MS. et al. measured RV thickness using automatic software at any site within the RV wall and reported an average RV thickness of 7 ± 2 mm in patients with HCM^4^. Nagata Y. et al. defined RV hypertrophy as RV maximal wall thickness > 5 mm; the average maximal RV thickness was 4.7 ± 2.3 mm in the total patient population and 7.8 ± 1.8 mm in patients with RV hypertrophy in their study. Based on these previous studies, we defined RV involvement as the maximum RV wall thickness of ≥ 7 mm. We presumed that this criterion is reasonable to minimize the possibility of false-positive and false-negative findings of RV involvement. Further large population and prospective studies are required to standardize the diagnostic criteria of RV involvement in HCM. Third, we could not include data of genetic testing for HCM and follow-up CMR due to the retrospective nature of the study. Information regarding genetic mutation and serial change in the RV phenotype orn CMR could provide more concomitant evidence of the mechanism of RV involvement.

In conclusion, RV involvement in patients with HCM is common. Patients with RV involvement showed more severe myocardial dysfunction of the LV and RV, suggesting that it can be considered an indicator of severe HCM. Furthermore, RV involvement and impaired RV longitudinal strain in HCM showed clinical significance related to adverse clinical outcomes.

## Methods

### Study population

A total of 346 patients who underwent both cardiac magnetic resonance (CMR) imaging and transthoracic echocardiography within 6 months of each other were screened in Yonsei University Cardiovascular Hospital in the Republic of Korea. Patients who had undergone septal myectomy, had combined heart disease leading to RV hypertrophy, or had significant pulmonary hypertension defined as RV systolic pressure (RVSP) > 50 mmHg were excluded. Finally, 256 patients were included for the analysis. The study was approved by the Institutional Review Board of the Yonsei University Health System (approval number: 4-2012-0655), and it complied with the ethical principles of the Declaration of Helsinki. The need for informed consent was waived by the local ethics committee of the Yonsei University Health System.

### Cardiac magnetic resonance imaging

**Figure 3:**
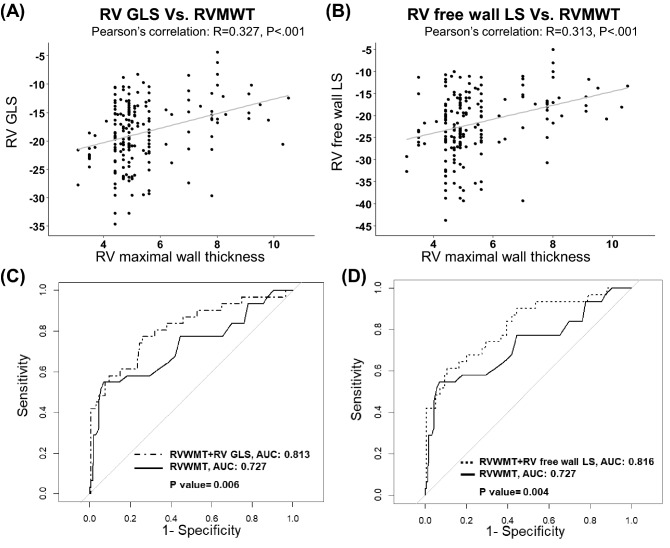
Scatter plot and Pearson correlation coefficient between **(A)** right ventricular maximal wall thickness (RVMWT) and RV global longitudinal strain (GLS) and **(B)** RVMWT and RV free wall longitudinal strain (LS). Comparison of receiver operating characteristic curves with corresponding areas under the curve to predict RV involvement between **(C)** RV wall thickness and RV wall thickness with RV GLS and **(D)** RV wall thickness and RV wall thickness with RV free wall LS.

All patients underwent CMR with a 3-T clinical scanner (Magnetom Trio Tim, Siemens AG Healthcare Sector, Erlangen, Germany). Electrocardiogram gated cine images were acquired in the short-axis views using retrospective echocardiography-gated balanced steady-state free precession true fast imaging with steady-state precession (TrueFISP) sequence with the following parameters: repetition time (TR), 3.3 ms; echo time (TE), 1.44 ms; flip angle, 50°, 25 phases; slice thickness, 8 mm; slice gap, 8 mm; acquisition matrix, 216 × 256 pixels; and field of view, 337 × 400 mm^2^. LV and RV LGE images were acquired 10 min after contrast injection (0.2 mmol/kg of a gadolinium-based contrast media) using a magnitude- and phase-sensitive inversion-recovery-prepared TrueFISP sequence, with the inversion time adjusted to null, thus representing the normal myocardium. Two expert radiologists, blinded to patients’ clinical data, analysed CMR images. All image analyses were performed using semi-automatic segmentation in the software (CMR42, Circle Cardiovascular Imaging, Calgary, Alberta, Canada). Additionally, the left and right ventricular volumes and ejection fractions (EFs) were measured from the cine images using semi-automatic segmentation in the software (CMR42, Circle Cardiovascular Imaging, Calgary, Alberta, Canada). LV (LVMWT) and RV (RVMWT) maximal wall thickness were measured as the greatest dimension at end-diastole measured manually in short-axis slices. RVMWT was measured in the segments of the RV free wall, as shown in Fig. 3A. The presence of LGE in LV and RV was also confirmed separately (see Fig. 3B). Pericardium and trabeculations were excluded from the assessment of wall thickness. RV involvement was defined as maximum RV wall thickness of ≥ 7 mm in any segments of the RV free wall.

### Echocardiography

Two-dimensional linear and volumetric measurements were obtained using standard methods^22,23^. LVMWT was determined at end-diastole from the parasternal short-axis view. RV wall thickness was measured at end-diastole below the tricuspid annulus at a distance approximating the length of the anterior tricuspid leaflet^24^. Obstruction of the LV outflow was defined as the peak pressure gradient of the LV outflow tract ≥ 30 mmHg on continuous-wave Doppler echocardiography at rest or by Valsalva manoeuvre. In a subgroup analysis, LV and RV mechanical function were evaluated in 190 patients using speckle tracking echocardiography (STE). LV and RV strain were assessed via STE analysis performed offline using customized software (EchoPAC PC; GE Medical Systems). Three consecutive cardiac cycles were recorded and averaged, and frame rates were set to 60–80 frames per second. LV global longitudinal strain (GLS) was obtained from the average of three standard apical views. RV GLS was defined as the average of the RV free wall and septal segments measured in standard focused RV view or apical four-chamber view using the software designed for LV measurements and adapted for the RV. RV free wall longitudinal strain (LS) was defined as the arithmetical average of three segments (base, mid, and apex) of the RV free wall (see Fig. 3C,D). Impaired RV free wall longitudinal strain was defined as > − 20%^23,25^.

### Outcomes

Primary outcomes were defined as composite of all-cause death, heart transplantation, and cardiovascular hospitalization during the follow-up period (median: 1153 days [interquartile range: 748–1372 days]). A cardiovascular hospitalization was defined as an unplanned cardiovascular event requiring admission for heart failure, angina, atrial or ventricular tachyarrhythmia, sudden collapse, stroke, or myocardial infarction. The clinical events were analysed by two researchers independently, and the occurrence of renal outcomes was decided with the agreement of both researchers.

### Statistical analysis

All continuous data are presented as mean ± standard deviation, and categorical data are expressed as numbers and percentages for each group. Interobserver agreement for the presence of RV involvement was calculated using Cohen’s kappa value. The significance of RV involvement on primary outcomes was analysed with multivariate Cox proportional hazard models and Kaplan–Meier curves. Correlation between RVMWT and parameters of LV and RV strain was calculated using Pearson’s correlation method. Diagnostic incremental values of the echocardiographic parameters identifying RV involvement were estimated using receiver operating characteristic (ROC) curves with corresponding areas under the curve (AUC). Comparisons between the AUC were conducted using DeLong's test^26^. All tests were two-sided, and statistical significance was defined as p < 0.05. All statistical analyses were performed with R statistical software (version 3.6.0; R Foundation for Statistical Computing, Vienna, Austria).

## Supplementary Information

Supplementary Table 1.

## Data Availability

All data used during this study will be available from the corresponding author if the request is rational.

## References

[1] Authors/Task Force members, Elliott, P. M. *et al.* 2014 ESC guidelines on diagnosis and management of hypertrophic cardiomyopathy: The task force for the diagnosis and management of hypertrophic cardiomyopathy of the European Society of Cardiology (ESC). *Eur. Heart J.* **35**, 2733–2779, 10.1093/eurheartj/ehu284 (2014). 10.1093/eurheartj/ehu284 25173338

[2] Gersh, B. J. *et al.* 2011 ACCF/AHA guideline for the diagnosis and treatment of hypertrophic cardiomyopathy: A report of the American College of Cardiology Foundation/American Heart Association task force on practice guidelines. Developed in collaboration with the American Association for Thoracic Surgery, American Society of Echocardiography, American Society of Nuclear Cardiology, Heart Failure Society of America, Heart Rhythm Society, Society for Cardiovascular Angiography and Interventions, and Society of Thoracic Surgeons. *J. Am. Coll. Cardiol.* **58**, e212–260, 10.1016/j.jacc.2011.06.011 (2011). 10.1016/j.jacc.2011.06.011 22075469

[3] Maron BJ (2018). Clinical course and management of hypertrophic cardiomyopathy. N. Engl. J. Med..

[4] Maron MS (2007). Right ventricular involvement in hypertrophic cardiomyopathy. Am. J. Cardiol..

[5] Nagata Y (2015). Right ventricular hypertrophy is associated with cardiovascular events in hypertrophic cardiomyopathy: Evidence from study with magnetic resonance imaging. Can. J. Cardiol..

[6] Wu XP (2019). Impaired right ventricular mechanics at rest and during exercise are associated with exercise capacity in patients with hypertrophic cardiomyopathy. J. Am. Heart Assoc..

[7] Shah JP (2018). Prevalence and prognostic significance of right ventricular dysfunction in patients with hypertrophic cardiomyopathy. Am. J. Cardiol..

[8] Mozaffarian D, Caldwell JH (2001). Right ventricular involvement in hypertrophic cardiomyopathy: A case report and literature review. Clin. Cardiol..

[9] Butz T (2008). Significant obstruction of the right and left ventricular outflow tract in a patient with biventricular hypertrophic cardiomyopathy. Eur. J. Echocardiogr..

[10] McKenna WJ, Kleinebenne A, Nihoyannopoulos P, Foale R (1988). Echocardiographic measurement of right ventricular wall thickness in hypertrophic cardiomyopathy: Relation to clinical and prognostic features. J. Am. Coll. Cardiol..

[11] Guo X (2017). The clinical features, outcomes and genetic characteristics of hypertrophic cardiomyopathy patients with severe right ventricular hypertrophy. PLoS ONE.

[12] Maron BJ, Maron MS (2016). Contemporary strategies for risk stratification and prevention of sudden death with the implantable defibrillator in hypertrophic cardiomyopathy. Heart Rhythm.

[13] Maron MS (2016). Contemporary natural history and management of nonobstructive hypertrophic cardiomyopathy. J. Am. Coll. Cardiol..

[14] Geske JB, Sorajja P, Nishimura RA, Ommen SR (2007). Evaluation of left ventricular filling pressures by Doppler echocardiography in patients with hypertrophic cardiomyopathy: Correlation with direct left atrial pressure measurement at cardiac catheterization. Circulation.

[15] Yang WI (2009). Left atrial volume index: A predictor of adverse outcome in patients with hypertrophic cardiomyopathy. J. Am. Soc. Echocardiogr..

[16] Melacini P (2010). Clinicopathological profiles of progressive heart failure in hypertrophic cardiomyopathy. Eur. Heart J..

[17] Kitaoka H (2011). Tissue doppler imaging and plasma bnp levels to assess the prognosis in patients with hypertrophic cardiomyopathy. J. Am. Soc. Echocardiogr..

[18] Tower-Rader A (2018). Prognostic value of global longitudinal strain in hypertrophic cardiomyopathy: A systematic review of existing literature. JACC Cardiovasc. Imaging.

[19] Finocchiaro G (2014). Prevalence and clinical correlates of right ventricular dysfunction in patients with hypertrophic cardiomyopathy. Am. J. Cardiol..

[20] D'Andrea A (2010). Right ventricular myocardial involvement in either physiological or pathological left ventricular hypertrophy: An ultrasound speckle-tracking two-dimensional strain analysis. Eur. J. Echocardiogr..

[21] Li, X. *et al.* Assessing right ventricular deformation in hypertrophic cardiomyopathy patients with preserved right ventricular ejection fraction: A 3.0-t cardiovascular magnetic resonance study. *Sci Rep* **10**, 1967, 10.1038/s41598-020-58775-0 (2020). 10.1038/s41598-020-58775-0 PMC7004999 32029853

[22] Mitchell C (2019). Guidelines for performing a comprehensive transthoracic echocardiographic examination in adults: Recommendations from the American Society of Echocardiography. J. Am. Soc. Echocardiogr..

[23] Lang RM (2015). Recommendations for cardiac chamber quantification by echocardiography in adults: An update from the American Society of Echocardiography and the European Association of Cardiovascular Imaging. Eur. Heart J. Cardiovasc. Imaging.

[24] Rudski, L. G. *et al.* Guidelines for the echocardiographic assessment of the right heart in adults: A report from the American Society of Echocardiography endorsed by the European Association of Echocardiography, a registered branch of the European Society of Cardiology, and the Canadian Society of Echocardiography. *J. Am. Soc. Echocardiogr.* **23**, 685–713; quiz 786–688, 10.1016/j.echo.2010.05.010 (2010). 10.1016/j.echo.2010.05.010 20620859

[25] Badano LP (2018). Standardization of left atrial, right ventricular, and right atrial deformation imaging using two-dimensional speckle tracking echocardiography: A consensus document of the EACVI/ASE/industry task force to standardize deformation imaging. Eur. Heart J. Cardiovasc. Imaging.

[26] DeLong ER, DeLong DM, Clarke-Pearson DL (1988). Comparing the areas under two or more correlated receiver operating characteristic curves: A nonparametric approach. Biometrics.

