# Supplementary material for: Prevalence, functional characteristics, and clinical significance of right ventricular involvement in patients with hypertrophic cardiomyopathy
Source: Sci Rep. 2020 Dec 14;10:21908. doi: 10.1038/s41598-020-78945-4 (PMC7736330; doi:10.1038/s41598-020-78945-4)
Supplement: Supplementary file 1 — Supplementary Table 1. [file 41598_2020_78945_MOESM1_ESM.doc]

**Prevalence, Functional Characteristics, and Clinical Significance of Right Ventricular Involvement in Patients with Hypertrophic Cardiomyopathy**

Jiwon Seo^1^, MD; Yoo Jin Hong^2^, MD, PhD; Young-Jin Kim^2^, MD, PhD; Purevjargal Lkhagvasuren^1^, MD; Iksung Cho, MD^1^; Chi Young Shim^1^, MD, PhD; Jong-Won Ha^1^, MD, PhD; Geu-Ru Hong^1*^, MD, PhD

^1^Cardiology Division, Severance Cardiovascular Hospital, Yonsei University College of Medicine, Seoul, Korea

^2^Department of Radiology, Research Institute of Radiological Science, Severance Hospital, Yonsei University College of Medicine, Yonsei University Health System, Seoul, South Korea

**Short title:** HCM with RV involvement

**^*^Corresponding Author:**

Geu-Ru Hong, M.D., Ph.D.

Division of Cardiology, Severance Cardiovascular Hospital, Yonsei University College of Medicine, 50-1 Yonsei-ro, Seodaemun-gu, Seoul, Republic of Korea 03722

Phone: +82-2-2228-8453, Fax: +82-2-2227-7742, E-mail: [GRHONG@yuhs.ac](mailto:GRHONG@yuhs.ac)

Supplementary Table 1. Baseline characteristics of study population

|  | Total patients  (n=190) | No RV involvement  (n=160) | RV involvement  (n=30) | P value^*^ |
| --- | --- | --- | --- | --- |
| **Demographic and clinical data** |  |  |  |  |
| Age, year ± SD | 52.5 ± 14.1 | 52.7 ± 14.2 | 50.9 ± 14.1 | 0.514 |
| Male sex, n (%) | 129 (67.9%) | 106 (66.2%) | 23 (76.7%) | 0.364 |
| BMI, kg/m^2^ ± SD | 24.8 ± 3.2 | 24.8 ± 3.1 | 24.5 ± 3.4 | 0.577 |
| Hypertension, n (%) | 141 (74.2%) | 119 (74.4%) | 22 (73.3%) | 0.999 |
| Diabetes mellitus, n (%) | 38 (20.0%) | 32 (20.0%) | 6 (20.0%) | 0.999 |
| Dyslipidemia, n (%) | 68 (35.8%) | 52 (32.5%) | 16 (53.3%) | 0.048 |
| Atrial fibrillation, n (%) | 33 (17.4%) | 23 (14.4%) | 10 (33.3%) | 0.024 |
| Familiar history of SCD, n (%) | 29 (15.3%) | 26 (16.2%) | 3 (10.0%) | 0.551 |
| ICD, n (%) | 11 (5.8%) | 6 (3.8%) | 5 (16.7%) | 0.019 |
| Syncope, n (%) | 22 (11.6%) | 17 (10.6%) | 5 (16.7%) | 0.523 |
| Beta blocker, n (%) | 89 (46.8%) | 77 (48.1%) | 12 (40.0%) | 0.536 |
| Calcium channer blocker, n (%) | 47 (24.7%) | 37 (23.1%) | 10 (33.3%) | 0.338 |
| RAS blocker, n (%) | 84 (44.2%) | 75 (46.9%) | 9 (30.0%) | 0.132 |
| Aspirin, n (%) | 46 (24.2%) | 34 (21.2%) | 12 (40.0%) | 0.049 |
| Statin, n (%) | 58 (30.5%) | 43 (26.9%) | 15 (50.0%) | 0.021 |
| **Echocardiographic data** |  |  |  |  |
| LVEF, % ± SD | 68.7 ± 8.5 | 69.0 ± 7.9 | 67.2 ± 11.4 | 0.420 |
| LAVI, ml/m^2^ ± SD | 42.2 ± 16.1 | 40.9 ± 15.1 | 49.4 ± 19.8 | 0.031 |
| RV systolic pressure, mmHg ± SD | 26.8 ± 7.2 | 26.4 ± 6.8 | 28.9 ± 9.3 | 0.170 |
| E/e’ ± SD | 15.2 ± 6.8 | 14.7 ± 6.7 | 18.0 ± 7.0 | 0.013 |
| LV maximal thickness, mm ± SD | 21.3 ± 4.6 | 20.6 ± 4.2 | 24.8 ± 5.4 | <.001 |
| RV thickness, mm ± SD | 4.3 ± 1.5 | 4.0 ± 0.9 | 5.9 ± 2.7 | 0.001 |
| LVOT obstruction, n (%) | 46 (24.2%) | 37 (23.1%) | 9 (30.0%) | 0.566 |
| **Cardiac MR data** |  |  |  |  |
| LV maximal thickness, mm ± SD | 21.7 ± 5.1 | 21.3 ± 4.8 | 23.8 ± 6.1 | 0.012 |
| RV maximal thickness, mm ± SD | 5.5 ± 1.9 | 4.8 ± 0.6 | 9.0 ± 2.4 | <.001 |
| LVEDV, ml ± SD | 143.8 ± 35.0 | 143.4 ± 33.7 | 146.4 ± 42.0 | 0.671 |
| LVESV, ml ± SD | 48.6 ± 21.0 | 47.3 ± 20.4 | 55.8 ± 23.3 | 0.049 |
| LVEF, % ± SD | 67.4 ± 9.0 | 68.2 ± 8.4 | 63.3 ± 10.7 | 0.005 |
| RVEDV, ml ± SD | 127.0 ± 34.8 | 127.3 ± 34.5 | 125.0 ± 37.0 | 0.750 |
| RVESV, ml ± SD | 50.6 ± 20.8 | 51.0 ± 21.1 | 48.4 ± 19.6 | 0.537 |
| RVEF, % ± SD | 61.1 ± 9.4 | 61.0 ± 9.2 | 61.7 ± 10.8 | 0.731 |
| LV LGE, n (%) | 179 (94.2%) | 149 (93.1%) | 30 (100.0%) | 0.292 |
| RV LGE, n (%) | 21 (11.1%) | 3 (1.9%) | 18 (60.0%) | <.001 |
| ^*^P value between patients with RV involvement and without RV involvement  RV, right ventricle; LV, left ventricle; BMI, body mass index; SCD, sudden cardiac death; RAS, renin-angiotensin system; LVEF, LV ejection fraction; LAVI, left atrial volume index; LVOT, left ventricular outflow tract; LS, longitudinal strain; GLS, global LS; MR, magnetic resonance; LVEDV, LV end diastolic volume; LVESV, LV end systolic volume; RVEDV, RV end diastolic volume; RVESV, RV end systolic volume; LGE, late gadolinium enhancement | | | | |
